# No involvement of alveolar macrophages in the initiation of carbon nanoparticle induced acute lung inflammation in mice

**DOI:** 10.1186/s12989-016-0144-6

**Published:** 2016-06-21

**Authors:** Shanze Chen, Renfu Yin, Kathrin Mutze, Youjia Yu, Shinji Takenaka, Melanie Königshoff, Tobias Stoeger

**Affiliations:** 1 Comprehensive Pneumology Center, Institute of Lung Biology and Disease, Helmholtz Zentrum München, Member of the German Center for Lung Research (DZL), Munich, Germany; 2 Department of Pathophysiology, West China School of Preclinical Sciences and Forensic Medicine, Sichuan University, Chengdu, 610041 Sichuan Province People’s Republic of China; 3 Department of Veterinary Preventive Medicine, College of Veterinary Medicine, Jilin University, Changchun, Jilin China

**Keywords:** Carbonaceous nanoparticles (CNP), Intratracheal instillation, Lung inflammation, Sterile inflammation, Alveolar macrophage, Alveolar epithelial cell, Neutrophils, Chemokines

## Abstract

**Background:**

Carbonaceous nanoparticles (CNP) represent a major constituent of urban particulate air pollution, and inhalation of high CNP levels has been described to trigger a pro-inflammatory response of the lung. While several studies identified specific particle characteristics driving respiratory toxicity of *low-solubility and low-toxicity particles* such as CNP, the major lung cell type, which initiates and drives that response, remains still uncertain. Since alveolar macrophages (AM) are known to effectively phagocytose inhaled particles and play a crucial role for the initiation of pulmonary inflammation caused by invading microbes, we aimed to determine their role for sterile stimuli such as CNP by profiling the primary alveolar cell compartments of the lung. We exposed C57BL/6 mice to 20 μg CNP by intratracheal instillation and comprehensively investigated the expression of the underlying mediators during a time span of 3 to 72 h in three different lung cell populations: CD45- (negative) structural cells, CD45+ (positive) leukocytes, and by BAL recovered cells.

**Results:**

Bronchoalveolar lavage (BAL) analysis revealed an acute inflammatory response characterized by the most prominent culmination of neutrophil granulocytes from 12 to 24 h after instillation, which declined to basal levels by day 7. As early as 3 h after CNP exposure 50 % of the AM revealed particle laden. BAL concentrations and lung gene expression profiles of TNFα, and the neutrophil chemoattractants CXCL1,-2 and-5 preceded the neutrophil recruitment and showed highest levels after 12 h of CNP exposure, pointing to a significant activation of the inflammation-evoking lung cells at this point of time. AM, isolated from lungs 3 to 12 h after CNP instillation, however, did not show a pro-inflammatory signature. On the contrary, gene expression analysis of different lung cell populations isolated 12 h after CNP instillation revealed CD45-, mainly representing alveolar epithelial type II (ATII) cells as major producer of inflammatory CXCL cytokines. Particularly by CD45- cells expressed Cxcl5 proved to be the most abundant chemokine, being 12 h after CNP exposure 24 (±11) fold induced.

**Conclusion:**

Our data suggests that AM are noninvolved in the initiation of the inflammatory response. ATII cells, which induced highest CXCL levels early on, might in contrast be the driver of acute neutrophilic inflammation upon pulmonary CNP exposure.

**Electronic supplementary material:**

The online version of this article (doi:10.1186/s12989-016-0144-6) contains supplementary material, which is available to authorized users.

## Background

Black carbon, the most strongly light absorbing-component of particulate matter (PM), is an air pollutant increasingly discussed to affect both human health and climate change [1]. For Western Europe, traffic seems to be the most important source and average exposures of 6.5 ug/m^3^ have been described for people in transport [2]. Black carbon is mainly present in the form of soot particles within the so-called ultrafine, nanoparticle fraction of anthropogenic air pollution, but in addition to the inhalation of combustion-derived nanoparticles, the workplace can also be an important site of exposure in the case of manufacture of carbon black. Due to its small size, inhaled carbonaceous nanoparticles (CNP) have been described to effectively penetrate deep into the lungs, deposit in the fragile alveolar region and might thus be more potent to induce health effects than lager particles [3–5]. Even that associations of specific sub-components of PM with adverse effects are difficult to detect for epidemiological studies, combustion-derived particles had been identified as an important component in driving adverse effects of PM already years ago [6, 7]. In this context the WHO report 2012 recognizes sufficient evidence of epidemiological studies for an association of daily variations in black carbon concentrations with short‑term changes in health (here: all‑cause and cardiovascular mortality, and cardiopulmonary hospital admissions), and suggest BC to be a better indicator of harmful particulate substances from combustion sources (especially traffic) than undifferentiated PM mass. We and others have used carbon nanoparticles of various sources, such as Printex 90 or Sterling V to examine the underlying toxicological mechanisms in animal and human studies [8–12]. While these CNPs exhibit difficulties in the aerosolization of agglomerated black carbon powders in the ultrafine or nanosized mode (<100 nm count median diameter), freshly, lab generated CNPs have been used for inhalation studies in mice [13, 14], rats [15], and man [16, 17]. Depending on the focus of interest and also the doses applied, these studies describe low grade pulmonary inflammation, trombogenicity, cardiovascular impairments and alterations in the peripheral blood leukocyte distribution upon CNP exposure.

With regard to the initiation event that leads to the observed exacerbations of cardiorespiratory effects caused by inhaled particles, the most-recognized hypothesis is the oxidative stress paradigm. According to this hypothesis the underlying pathophysiological mechanism of particle toxicity for so called *low-solubility and low-toxicity particles (LSLTP)* is dependent on particle triggered oxidative stress and subsequent inflammation [18, 19]. The most prominent feature for this innate immune response is the recruitment and activation of granulocytes, specifically neutrophils, to the site of stimulus, here the site of pulmonary particle deposition [20, 21]. For LSLTP such as titanium dioxide, polystyrene or carbonaceous nanoparticles (CNP), the particle induced pulmonary inflammatory effect, assessed as number of neutrophils accumulated in the airspace of the lungs, is predominantly driven by oxidative surface properties of the pulmonary deposited particle [22]. As consequence and due to their high specific surface area, nanoparticles have been shown to be more inflammogenic than fine particles of identical chemical composition [20, 23, 24].

However, which cell type upon particle deposition finally initiates the inflammatory cascade remains obscure. Broadly speaking the alveolar compartment, as main site of nanoparticle deposition and retention, consists of three different cell types which line the alveolar surface and are thus directly in contact with the deposited particles: type I (ATI) and type II (ATII) alveolar epithelial cells and in the epithelial lining fluid nestled alveolar macrophages (AM). Even that a ‘three cell model’ is oversimplified, and various other immune relevant cell types such as dendritic cells, mast cells, interstitial macrophages and fibroblasts will have to be considered [25], we like to start from this simplistic view and focus here at the alveolar surface, which is likely bearing the highest particle burden upon CNP inhalation. AT1 cells cover 98 % of the alveolar surface [26, 27], ATII cells secrete surfactant, maintain the fluid balance and have been described as defender of the alveolus [28]. The tissue resident AM are known for their effective uptake of deposited particles and also nanoparticles [29], and mediate acute lung inflammation and resolution in many disease conditions [30].

The recruitment of neutrophils to the site of injury is generally initiated by the binding of the neutrophil chemoattractants CXCL1, -2 and -5 to the neutrophil chemokine receptor CXCR2 [20]. CXCL1 can be expressed by macrophages, neutrophils and epithelial cells during the inflammatory response [31]. CXCL2, also referred to as MIP2α (macrophage inflammatory protein 2-alpha), in contrast is mainly secreted by monocytes and macrophages [32]. CXCL5, also known as ENA-78 (epithelial-derived neutrophil-activating peptide 78), is a small cytokine and mainly expressed by epithelial cells [33, 34]. Till today no specific signaling receptor or cell type recognizing sterile particles such as CNP or other LSLTP has been described and related to the evoked inflammatory response in the lung. Even that promising studies have recently uncovered the activation of e.g. epidermal growth factor (EGF) receptor [35] or pattern recognition receptors by different nanoparticles [36], it is still unclear how relevant this interaction may act as initial trigger for the inflammatory response, caused by inhaled LSLTP particles. Since our mechanistic understanding of the early phase of the cellular course of events from particle deposition to neutrophil accumulation in the alveolar airspace of CNP exposed lungs remains elusive, we might be tempted to compensate this gap by employing a well-established mode of action, such as the one known for neutrophil recruitment triggered by bacterial infection. Here pathogen-associated molecular pattern are recognized by specific pattern recognition receptors and tissue macrophages are considered crucial sensors for pathogen-invasion and cell damage. A similar macrophage based understanding exists for the pulmonary toxicity of quartz particles and fibers. Particle phagocytosis of needle like shapes or particular crystals can combined with the material specific high surface reactivity cause lysosomal destabilization, which sensed by the NALP3 inflammasome, leads to the release of the pro-inflammatory master cytokine IL-1β from particle laden macrophages [37, 38]. In addition to that, IL-α had been described as a alarmin cytokine, upstream of IL-1β, which is released from AM already in the first hours after silica particle deposition [39]. None of these activation mechanisms have so far been observed for pyrogen-free, spherical carbon nanoparticles, and a specific role of AM in sensing these particles as a danger pattern remains uncertain.

Several, but mainly in vitro based studies, suggest a central function of the respiratory epithelium for the inflammatory response caused by carbonaceous nanoparticles. In this context it has been described that fine PM induces amphiregulin secretion by bronchial epithelial cells [40]. Our previous CNP inhalation study also detected induced pulmonary expression of this epidermal growth factor (EGF)-like molecule [13]. In vitro, high doses of CNP have been shown to trigger, apoptosis and proliferation of ATII cells via pathways using EGF-receptor signaling [41]. The amphiregulin EGF-receptor interaction may also cause MAP-kinase activation and thus function as a pro-inflammatory feedback loop in the lung epithelium [42]. But again, the in vivo relevance of all these epithelial or macrophage pathways for the initiation of CNP triggered inflammation is not confirmed.

Therefore, in this study, we aimed to identify the pulmonary cell type, which initiates the inflammation and produces the neutrophil chemoattractants upon CNP exposure. Since the majority of inhaled nanoparticles are rapidly incorporated by AM, thereby posing the highest cellular dose to these cells, and since activated macrophages are in general well known for their extreme cytokine production capability, we hypothesize that alveolar macrophages play a key role for the initiation of CNP induced lung inflammation. In order to address this question, we performed a time course analysis to identify the onset of the inflammatory response, isolated AM by Bronchoalveolar lavage (BAL), CD45+ lung tissue leukocytes and CD45- lung epithelial cells from the CNP instilled mice and examined the expression of inflammatory genes and neutrophil chemoattractants. Much to our surprise however, a comparison of the gene expression profiles of the three isolated cell populations uncovered an essential role of the alveolar epithelium, most likely type II (ATII) cells, for the production of the neutrophil chemoattractant at the onset of CNP induced inflammation, but not for the particle laden AM. Also no activation of alveolar macrophages preceding the cellular inflammation could be detected during the first 3 to 12 h after exposure.

## Results

We used a single dose of 20 μg/mouse of carbon nanoparticles (CNP) generated by spark discharge from graphite electrodes to induce an aseptic, acute neutrophilic inflammatory response in the lungs of healthy C57BL/6 mice by intratracheal instillation (IT). Particle dispersions where characterized by a mean agglomerate size of 190 nm (DSL) consisting of primary carbon particles of 7–12 nm (Additional file 1: Figure S1).

**Fig. 1:**
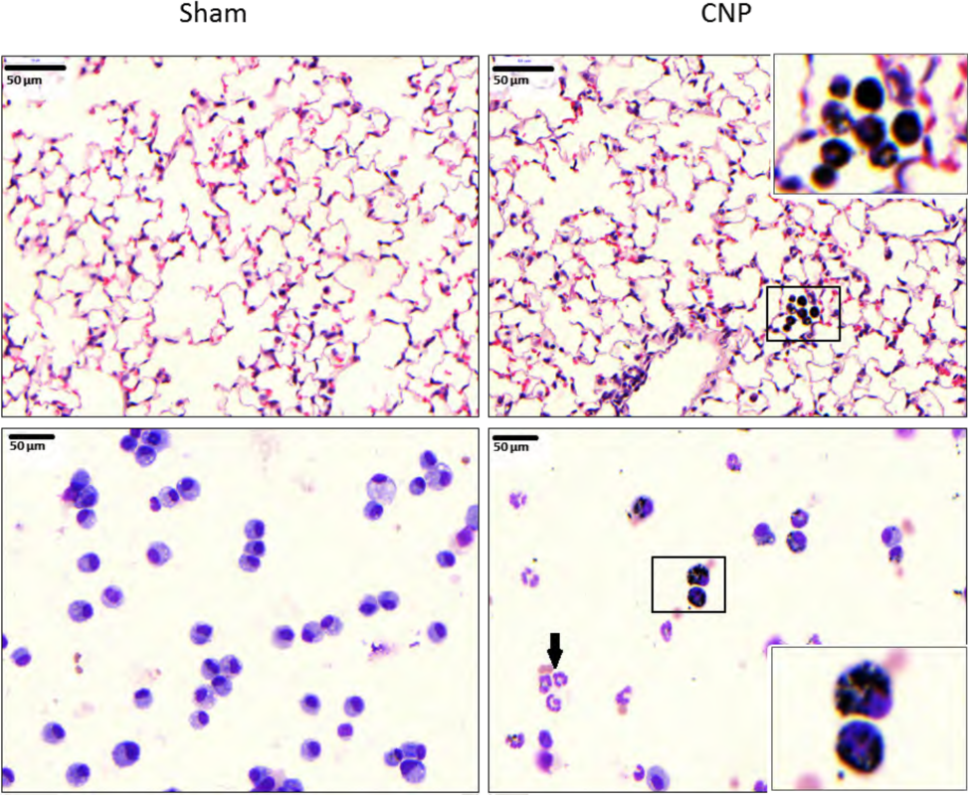
CNP agglomerates are engulfed by alveolar macrophages. The histological examination of hematoxylin and eosin stained lung sections (*upper panel*) reveals no detectable changes in the lung architecture, 24 h after sham and CNP instillation. A spotty accumulation of particle laden alveolar macrophages can be observed in the alveolar region of CNP treated lungs. Cytospin analysis of May Grunwald stained BAL cell (*lower panel*) underlines this finding and also indicates the inflammatory response by accumulation of neutrophilic granulocytes (*black arrow*). A magnification of particle laden macrophages, shown in the framed sections is illustrated at the upper (histology) and lower (BAL cells) right corner of the respective image

In comparison to sham treated controls, the IT delivery of 20ug CNP induced no detectable histological alterations in the epithelial architecture of the lungs within 24 h after instillation. However, particle laden alveolar macrophages (AM) can be detected in the alveoli and even more prominent on cytospin preparations of by BAL recovered cells from CNP treated lungs (Fig. 1). The appearance of particle laden AM was accompanied by an accumulation of polymorphonuclear neutrophils (PMN) 24 h after CNP IT. No particle agglomerates could be detected in structural cells, such as ATII cells.

### Time course response of BAL cells

**Fig. 2:**
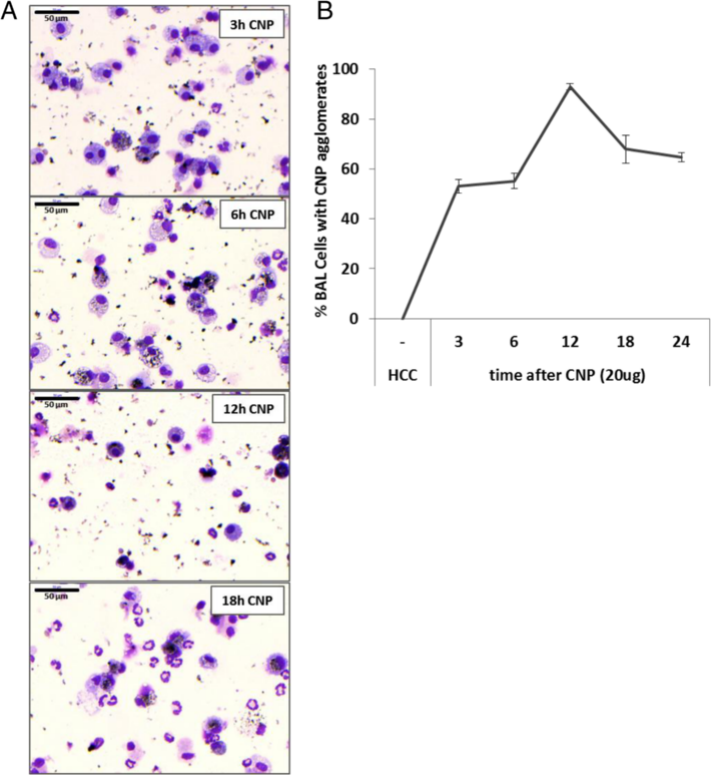
Time course of CNP agglomerate uptake. The amount of by BAL recovered cells showing inclusions of CNP agglomerates was quantified by the examination of cytospins from mice 3 to 24 h after particle instillation. Particle uptake was exclusively observed for macrophages but and no agglomerates we observed in polymorphonuclear, neutrophilic granulocytes (**a**). Free, not cell-associated particle agglomerates are predominantly detected at the early time points, till 12 h after exposure. Figure 2b shows the time course of the particle uptake given as the percentage of particle laden macrophages

We first performed a time course analysis to determine the starting point of the onset of the inflammatory response on the cellular as well as the molecular level. Accordingly, mice were sacrificed 3, 6, 12, 18, 24 h and 3 or 7 days past a single IT dose of 20 μg CNP, and BAL was analyzed for the influx of inflammatory cells and cytokines, and by qPCR for pro-inflammatory gene expression in lung homogenates. Counting the frequency of BAL cells with visible inclusions of back particle agglomerates (CNP) revealed a rapid increase of particle laden macrophages from 50 % already 3 h, to 90 % 12 h after CNP exposure. No CNP uptake was detected for neutrophils and free agglomerates were virtually absent for time points later than 12 h after CNP exposure (Fig. 2).

**Fig. 3:**
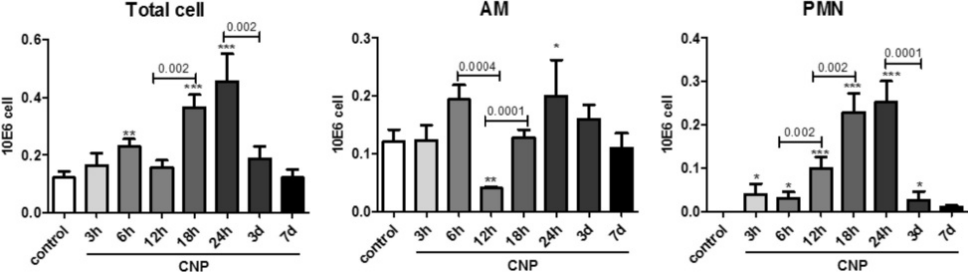
BAL cell numbers time course analysis reveals most dynamic PMN accumulation between 12 and 18 h after CNP exposure: The numbers of total BAL cells, alveolar macrophages (AM) and neutrophils (PMN) were determined by BAL cell differentiation from cytospin preparations of mice treated with 20 μg CNP for 3, 6, 12, 18, 24 h, and 3 or 7 days and compared to untreated controls. Most notable changes were observed for PMNs which are almost absent in controls (<1 %) but reach greatest numbers 18 and 24 h after exposure. Values are given as mean ± SEM, *n* = 5–10, asterisks represent significance as compared to control group with * *p* < 0.05, **p < 0.01 and ***p < 0.001. BAL lymphocyte numbers were below 1 % of total BAL cells in all groups (data not shown)

As depicted in Fig. 3, BAL cell differentiation revealed an acute, neutrophilic pulmonary inflammation, where neutrophilic granulocytes (PMNs) represent the most significant cell accumulation with 0.25 × 10^6^ PMNs 24 h after CNP instillation. Nevertheless, also 3 h after CNP, the first time point analyzed, a smaller number of 0.04 × 10^6^ PMNs could be detected, whereas PMNs were basically undetectable in untreated controls (below 1 %; <2 × 10^3^ cells). The increase in BAL PMN numbers was most dynamic from 12 to 18 h after treatment, with a significant increase from 0.10 to 0.23 x10^6^ PMNs within 6 h. Lymphocyte numbers were nearly undetectable (<1 % of total BAL cells) and macrophages showed a noticeable decline at the 12 h time point and an increase after 24 h only. The acute inflammatory response resolved over time, reaching BAL neutrophil levels similar to baseline by day 7.

### Time course response of neutrophil chemoattractants in BAL and lung homogenate

**Fig. 4:**
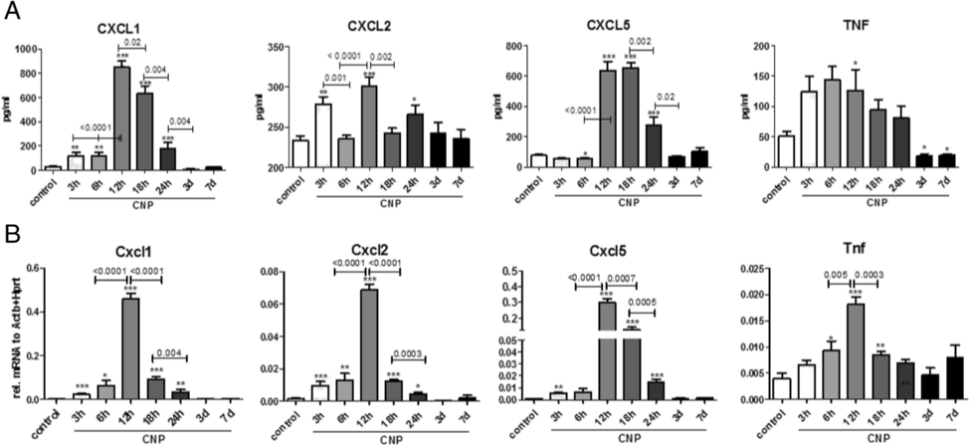
Time-course analysis of cytokine release and gene expression demonstrates most significant expression inductions from 6 to 12 h after CNP exposure: Concentrations of CXCL1,-2,-5 and TNFa cytokines were quantified by ELISA in BAL fluid from mice 3 h to 7 days after CNP exposure (**a**). Highest levels were detected at 12 h after CNP. Similarly, gene expression of Cxcl1, Cxcl2, Cxcl5 and Tnf determined by qPCR in whole lung tissue revealed the most notable expression inductions from 6 h to 12 h after treatment (**b**). Relative expressions are provided, normalized to Hprt and Actb. All values are given as mean ± SEM, *n* = 5–10, asterisks represent significance as compared to control group with *p < 0.05, **p < 0.01 and ***p < 0.001

Since the chemotactic recruitment of PMNs from the blood to the airspace is driven by chemokines released from different resident alveolar cells [25, 43], we next studied BAL levels of CXCL1,-2, and-5 as well as those of the well-known pro-inflammatory master cytokine TNFα during the inflammatory time course. As displayed in Fig. 4a, BAL concentrations of CXCL1, CXCL5 and TNFα showed a similar release pattern, with highest levels 12 h after CNP exposure. CXCL2 exhibited a more biphasic response, with highest levels at 6 and 12 h after treatment. In contrast to BAL PMN numbers, all investigated cytokine levels had dropped to baseline already by day 3, and TNFα concentrations were falling even below that of controls. With regard to the escalation of the BAL cytokine concentrations at 12 h after CNP, CXCL1 and CXCL5 levels actually multiplied by a factor of 30 and eight respectively, whereas TNFα and CXCL2 levels were as compared to controls induced only less than threefold. CXCL1 and-5 reached highest absolute BAL concentrations, with 850 and 640 pg/ml respectively, suggesting these two cytokines to be of major importance for the particle exposure triggered PMN recruitment to the airspace. Moreover, a significant increase in gene expression in lung homogenates for Cxcl1,-2,-5, and to a lower extent also for Tnf was detected by quantitative RT-PCR from 6 h to 12 h after CNP instillation (Fig. 4b). Cxcl and Tnf expression levels returned to baseline by day 3. We therefore consider the period from 6 h to 12 h after CNP instillation as the most critical phase for the cellular and transcriptional activation of the respective lung cells initiating the inflammatory response.

### CNP instillation has no impact on the inflammatory status of alveolar (BAL) macrophages

**Fig. 5:**
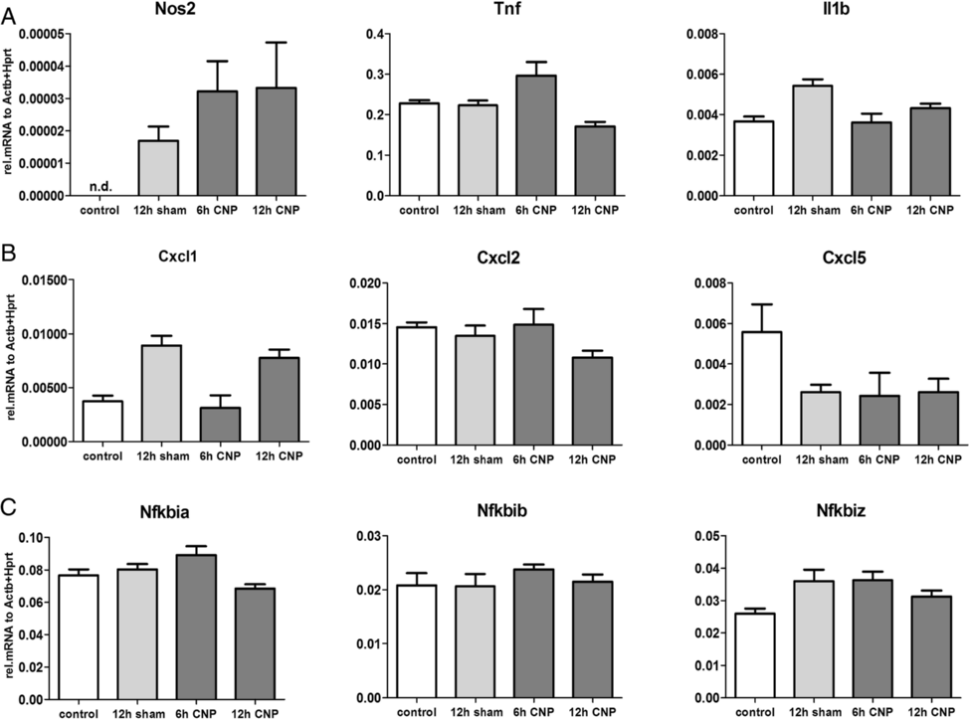
No effect of CNP IT on pro-inflammatory activation status of alveolar macrophages: Alveolar macrophages were isolated by BAL from mice 12 h after water (sham) or 6 and 12 h after CNP IT. Gene expression of the macrophage activation markers Nos2, Tnf and Il1b (**a**), the chemokines Cxcl1, Cxcl2 and Cxcl5 (**b**) and the NF- kB signaling pathway genes Nfkbia, Nfkbib and Nfkbiz (**c**) are presented relative to Actb + Hprt. Values are given as mean ± SEM, *n* = 4–5. (n.d.: not detected)

Since AM rapidly and effectively incorporate high burden of alveolar deposited CNPs (Fig. 2) and can therefore be considered to bear the highest cellular CNP dose, we next examined the activation status of these cells in dependence of the particle exposure. AM were isolated from mice by BAL either 3 and 12 h after sham treatment, or 3, 6 and 12 h after CNP IT and processed for qPCR. Expression analysis of the genes related to classical pro-inflammatory macrophage activation, Nos2 (nitric oxide synthase 2, inducible), Tnf and Il1b (Fig. 5a, and Additional file 1: Figure S5A) showed no exposure related changes. Also the Cxcl genes which showed significant upregulation in lung homogenates at 12 h after CNP treatment (Fig. 4b), revealed unresponsive in BAL AMs, 3, 6 and 12 h after CNP IT (Fig. 5a, and Additional file 1: Figure S5A). Noteworthy, under control conditions Cxcl1,-2 and-5 showed higher expression levels in BAL macrophages as compared to lung tissue extracts, but 12 h after CNP, total lung expressions exceeded those of AMs by magnitudes. In addition expression of the pro-inflammatory NF-kB pathway responsive genes Nfkbia, Nfkbib, and Nfkbiz (nuclear factor of kappa light polypeptide gene enhancer in B cells inhibitor alpha,-beta,-zeta) [44] did not show any change upon particle challenge in BAL AMs (Fig. 5c and Additional file 1: Figure S5). In contrast to the CNP exposure, instillation of endotoxin (0.1 μg LPS) caused a vigorous activation of the isolated BAL macrophages 12 h after, resulting in the well-known classical activation profile (Additional file 1: Figure S5). Altogether these data support the notion that the population of BAL isolated macrophages is not activated by particle instillation and thus does not seem to contribute to the inflammatory CNP response of the lung.

### Alveolar epithelial cells are a major producer of neutrophil attracting chemokines early after CNP treatment

To better understand which lung cells finally accomplish the pro-inflammatory gene expression profile observed 12 h after CNP exposure (Fig. 3b), we divided the lung homogenates of sham and CNP exposed mice in three different cell populations: CD45- (negative) structural lung cells, CD45+ (positive) lung leukocytes, and by BAL recovered cells (here not differentiated in adherent macrophages and non-adherent leukocytes). Cell purification was confirmed by expression profiling for the cell specific markers Cd68 (macrosialin) for macrophages. CD45- (negative) structural lung cells were almost exclusively (>95 %) composed of alveolar epithelial type II (ATII) cells, which was confirmed by Aqp1 (aquaporin 1) transcript level as well as immunostaining for- proSFTPC (pro surfactant protein C), cytokeratin (both positive) and α-Sma (α-smooth muscle actin), CD31, CD45, T1α (negative) (Additional file 1: Figure S2 and S3).

**Fig. 6:**
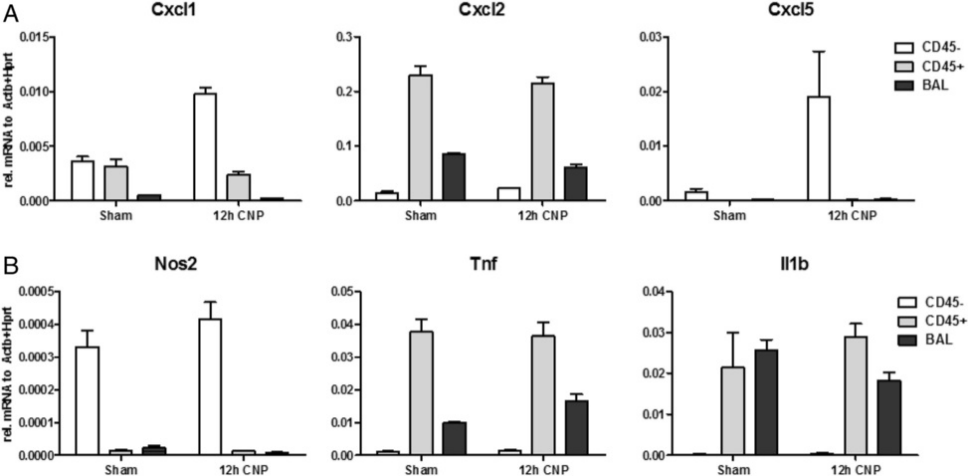
Alveolar epithelial cells are the main producers of neutrophil attracting chemokines: CD45- (alveolar epithelial cells), CD45+ leukocytes and total BAL cells were isolated from mice 12 h after the treatment with water (sham) or CNP. Gene expression analysis is shown for the chemokines Cxcl1, Cxcl2 and Cxcl5 (**a**), and the macrophage activation marker Nos2, Tnf and IL1b (**b**). Expression levels are given relative to Actb + Hprt. Results show means and SEM out of three replicas and the expression pattern is representative for four independent experiments

Comparative gene expression profiling highlighted that only ATII (CD45-) cells, but not CD45+ lung leukocytes or BAL cells, depict prominent induction of Cxcl1 and-5 mRNA levels. In fact, from the populations covered by our study, the main expression of Cxcl1 and-5 can be allocated to ATII cells, whereas Cxcl2 is predominantly expressed but not CNP-induced by lung and BAL leukocytes (Fig. 6a). Neither of the macrophage activation markers or pro-inflammatory master cytokines Nos2, Tnf and Il1b showed a conclusive expression profile supporting the inflammatory activation of CD45+ lung or BAL leukocytes (Fig. 6b and Additional file 1: Figure S4). To identify inflammatory activated cells also in situ, we applied a NF-kB1-GFP reporter mouse strain to visualize cell activation by anti-GFP immunohistochemistry in lung sections and BAL cell cytospin preparations (Additional file 1: Figure S6). While no cellular GFP accumulation could be detected in samples from controls or CNP (12 h) exposed mice, strong anti-GFP staining was observed for epithelial cells and macrophages of the LPS positive control (12 h).

In summary our results suggested a pivotal role of structural lung cells, in particular ATII cells, for the production and release of neutrophil attracting CXCL chemokines within the acute inflammatory response after CNP instillation. Alveolar macrophages in contrast seem not involved and do not get activated within the first 12 h after CNP exposure, despite of their prominent particle loading.

## Discussion

The inflammatory response is governed by a series of well-orchestrated intracellular and extracellular signaling events that control the expression and release of immune mediators, such as pro-inflammatory cytokines and chemokines [45]. One of the most important parameter and characteristics of acute inflammation is the migration of leukocytes to the site of injury. Particularly the accumulation of neutrophil granulocytes represents an essential defense strategy of the innate immune system and is recognized as the hallmark of acute inflammation [46]. The aim of the present study was to gain insight into the initiation of inflammation trigged by the inhalation of low-solubility and low-toxicity particles such as carbonaceous nanoparticles; more particularly we ask the question which cell type in the periphery of the lung may be the first activated and thus initiate the inflammatory response upon pulmonary particle deposition. In particular we raised the hypothesis that alveolar macrophages, the major phagocytes of the alveolus, get rapidly activated upon particle uptake and as consequence stimulated to express inflammatory mediators to kick-start the local inflammatory immune response.

We have chosen well-defined, lab-created carbon nanoparticles (CNP), which according to our previous work trigger significant lung inflammation within 24 h after instillation, at a moderate dose of 20 μg per mouse [12, 47]. We consider these particles as well suited representative for the so called ‘low-solubility and low-toxicity particles’ and have characterized them as virtually pure carbon particles without a considerable content of bio-active organic carbon compounds [48, 49], or endotoxin. The latter was confirmed by the absence of any inflammatory response in experiments with different murine macrophage cell lines, known for their endotoxin sensitivity (data not shown), and most importantly also by the lack of any pro-inflammatory CNP triggered macrophage stimulation in the current study. We have further chosen intratracheal instillation (IT) as delivery, superior to the more natural route of inhalation, because of the ease of dosimetry and the definite time point of pulmonary dose deposition as a result of the bolus IT delivery. In a previous CNP inhalation study we had in this context detected a bi-phasic gene expression response, characterized by an initial stress response phase during the first 4 h of CNP inhalation, followed by the inflammatory phase in the following 20 h [13]. This response pattern may be seen as a consequence of a gradually increasing dose accumulation combined with proceeding cellular stress and activation processes.

In the present study we first set out to define the time point of the onset of the cellular inflammatory response. BAL cell differentiation clearly indicated the main influx of PMNs into the alveolar airspace, to take place between 12 and 18 h after CNP IT, with the point of culmination around day 1 (Fig. 3). The neutrophilic inflammation showed a transient nature, and was completely resolved till day 7, which matches well with the absence of any histological changes or signs of sizeable lung tissue injury, which otherwise would decelerate the resolution process. A distinct expression and release of neutrophil attracting CXCL chemokines, accompanied and preceded the inflammatory recruitment of PMNs, in a way that Cxcl1,-2 and-5 gene expression increased most rapidly from 6 h to 12 h after the particle challenge (Fig. 4b) and yielded highest cytokine concentrations around 12 to 18 h after treatment. The 12 h time point showed also the most prominent activation of the NF-kB1 pathway in lung homogenates, indicated by electrophoretic mobility shift assay analysis of the specific recognition sequence for NF-kB, and the maximal expression of the NF-kB responsive genes Nfkbib, and Nfkbiz (data not shown).

This raises the question of which cells do actually get activated to drive the inflammatory response and generate the cytokine boost observed 12 h after CNP exposure. The most obvious candidate for the cell type which triggers the inflammation response to pulmonary particle deposition may be seen in the alveolar macrophage (AM). We have previously shown in rats, that already after a 6 h gold nanoparticle (14 nm) inhalation, particles were found in 94 % of the BAL AMs directly after the exposure period [29]. Accordingly also in the present study, carbon black laden AM were detected in the lungs already 3 h after CNP instillation, pointing to the highest cellular dose experienced by these tissue resident phagocytes. In view of the well-described potency, and necessity of AMs to respond to pathogens and to kick-off the local defenses against infection [50–52], we hypothesized that the massive contact with particles would induce an inflammatory activation of these cells. In this context, Brown et al. [53] showed the activation of rat AMs in vitro, by increased intracellular calcium, AP-1 transcription factor activation and TNFα production, all triggered by high doses of a very similar type of 14 nm CNP (Printex 90). So far however, no clear in vivo evidence was presented for the direct activation of AMs by pulmonary exposure to pure carbon particles.

Our expression analysis of by BAL recovered AMs from lungs 3, 6 and 12 h after CNP treatment, clearly contradict with the concept of AMs as the cellular inflammation trigger. In fact, AMs from CNP instilled mice showed in comparison to sham exposed or untreated controls, no sign of inflammatory activation. The expression of the major transcriptional markers for classical (M1) macrophage activation, Nos2 and Tnf showed no exposure related change, neither 3, nor 6, or 12 h after CNP treatment, nor was any other of the pro-inflammatory genes investigated such as Il1b, Cxcl1,-2,-5 or Nfkbia,-b, and –z, induced by the CNP treatment. Notwithstanding, all these M1 marker genes exhibited a significant induction within 12 h after a moderate endotoxin stimulation (Fig. 5). Other activation markers such Il6 or Ccl2 and also the most prominent factor Arg1 for alternative (M2) activation remained unchanged in AMs 12 h after CNP treatment.

On the basis of the total lack of pro-inflammatory responses in BAL AMs we had to reconsider our hypothesis of AMs being the alveolar cell population initiating the inflammatory stimulation. We therefore expanded our analysis to different primary cell isolates of the lung periphery from CNP and sham treated mice, to identify the cell type showing the most obvious inflammatory expression profile. From lavaged lungs we separated cells into CD45- lung epithelial cells and CD45+ lung leucocytes, and by lavage recovered BAL leucocytes. Immunohistochemical characterization revealed CD45- cells to be largely alveolar epithelial type II (ATII) cells (>95 %). This characterization completely agrees to previous characterizations of this way prepared lung cells [54–56] In fact this population showed the most robust induction of Cxcl1 and-5 gene expression 12 h after CNP exposure in relation to sham exposed lungs (Fig. 6a). The data thus suggests that ATII cells are an important source for the CXCL1 and-5 cytokine release observed 12 and 18 h after particle treatment (Fig. 4a). Noteworthy, CXCL1 and-5 were the most abundant chemokines identified, and were found to be released at concentrations of 600–900 pg/ml BAL fluid. In the contrary neither CD45+ lung leukocytes, nor BAL cells showed any sign for an inflammatory stimulation and marker genes like Cxcl2, Tnf, Nos2 and IL1b remained basically unchanged at the time point of investigation (Fig. 6b). This lack of inflammatory activation of total BAL cells after 12 h matched well with that, described before for BAL macrophages 3, 6 and 12 h after treatment, and supports the conclusion that AMs do not contribute to the initiation of the inflammatory response under sterile conditions. This of course was in sharp contrast to the by bacterial endotoxin provoked inflammation, were macrophages where characterized by abundant NFkB1 activation (Additional file 1: Figure S6) and pro-inflammatory gene expression (Additional file 1: Figure S5). It should be noted at this point, that the BAL cell population analyzed for Fig. 6 contains for the 12 h post CNP IT group already a great number of granulocytes (see Fig. 3) and the expression profiles do therefore not completely match with those shown in Fig. 5 for purified alveolar macrophages (see Additional file 1: Figure S4 for details). Hence the exposure related increases in Tnf and Il1b in BAL cells are likely attributable to leukocytes not present after purification.

Our observations, seem contradicting with the well described ability of AMs to produce a great number of pro-inflammatory cytokines upon the phagocytosis of environmental particles, a process caused by classical activation of these cells [19]. Also carbon black particles had been reported to generate ROS, cause a transient increases of intracellular calcium, and subsequently lead to the expression and release of TNFα in rat AM via activation of NF-KB and AP1 [53]. Therefore, our initial expectation was that lung macrophages contribute to the production of pro-inflammatory cytokines and chemokines to recruit circulating neutrophils to the alveolar space. In this context, we also exposed primary AMs and cells of the murine alveolar macrophage cell line MH-S to CNP in vitro, at doses up to 100 μg/ml. In contrast to Browns finding, we could not detect any induction of TNFα transcription or protein release. Yet exposure of MH-S cells to ambient PM_2.5_ effectively caused TNF expression and release, as did LPS [57]. The different outcomes for the similar carbon black particles might eventually be related to the different doses used. We however think that in vitro doses beyond 100 μg/ml medium are too excessive and likely to cause artefacts.

In summary, although that particle uptake in CNP exposed lungs can clearly be observed by the rapid accumulation of particle laden AMs (Fig. 2) these resident lung cells do not react to the CNP exposure with a classical pro-inflammatory activation during this initial phase, preceding the cellular inflammation (3–12 h). In contrast to this finding for insoluble, pure carbon particles, ambient PM samples are known for their potency to trigger the release of pro-inflammatory cytokines such as TNFα and IL-6 from exposed AMs [57], and this cytokine response is assumed to be crucial for the subsequent stimulation of the epithelium [58] or even cardiovascular effects beyond the lungs [59]. In our study CNP IT also induced highest levels of BAL IL-6 concentrations with up to 800 pg/ml 12 h after treatment, in comparison to levels below 10 pg/ml in controls, but again no exposure related change of Il6 gene expression could be detected in macrophages, neither in CD45+ nor in BAL cells at the same time point of investigation. Due to contaminations in ambient PM samples, such as airborne bacterial endotoxin or reactive metallic compounds, the described PM triggered induction of the pro-inflammatory cytokine release for AMs may however differ from that driven by pure carbon particles. On a related note, the initiation of CNP triggered inflammation seems also independent from the alarmin release mechanism, recently uncovered for crystalline silica particles [39]. In their study the authors describe the release of the tissue damage alarmin IL-1α from AM directly after pulmonary silica particle deposition. Opposed to this, no change in IL-1α BAL-levels could be detected in the CNP instilled lungs up to 24 h after exposure (data not shown), again arguing for a different kind of trigger for pure carbon particles.

Seong and Matzinger discuss in their highly stimulating review paper [60], how hydrophobic portions of exogenous particles or molecules can serve as ligands for different pathogen or damage associated molecular pattern such as Toll-like receptors and thereby induce an inflammatory response. From that view one might expect that especially pure carbon particles, characte rized by high hydrophobicity such as the here investigated CNP, should trigger such an ancient innate immune responses via the activation of respective damage associated molecular pattern (DAMP) receptors. First in vitro evidence for this interaction could be provided by exposure of splenocytes, to gold nanoparticles of different hydrophobicity [36]. Counter-intuitively and in contrast to this appealing hypothesis however our results deny an early exposition of DAMPs on the alveolar surface, as these motives would certainly cause immediate macrophage activation which we detected for LPS (Additional file 1: Figure S5 and S6), but not CNP IT. It is possible that the lung lining fluid and the thereby generated nanoparticle corona plays a protective role.

On the one hand we had to exclude AMs and probably even lung leukocytes (CD45+ cells) as the driver and initiator of CNP triggered neutrophil recruitment, on the other hand our data support that alveolar epithelial cells, and in particular the ATII cells play the crucial role in the CNP induced inflammatory process. Besides secretion of surfactant, ATII cells have also been shown to sense invading pathogens to produce antimicrobial products and amplify the inflammatory response by secretion of cytokine and chemokines [61, 62]. Particularly for nanoparticles, the alveolar epithelial layer has been described to accomplish uptake and translocation of deposited materials [63, 64]. For agglomerated carbonaceous particles this process might however be not effective enough [65] to a detection of epithelial internalized CNP agglomerates by light microscopy (Fig. 1). The fact that particularly CXCL5, also called epithelial-derived neutrophil-activating peptide 78 (ENA-78), is a specifically from inflammatory epithelial cells released chemokine, and showed highest expression levels 12 h after CNP instillation (Fig. 4), clearly indicates an early inflammatory activation of the epithelium by particle cell interactions. Unfortunately an immunohistological in situ detection of NF-kB driven cell activation failed in our hands for CNP exposed lungs, and only approved significant AM and alveolar epithelial NF-kB activation for LPS treated mice. Nevertheless, there is good evidence for both cell types ATI and -II to express and release CXCL5 under septic stimuli [33, 66, 67], but which cell type is the major source for sterile or nanoparticle triggered inflammation has after all not yet been elucidated.

In our study, the inflammatory activation of the epithelium showed a transient nature and resolved rapidly, in a way that Cxcl5 gene expression declined by a factor of 20 from 12 h to 24 h after CNP treatment (Fig. 4b). The time-course of maximal epithelial inflammatory activation from 6 to 12 h after CNP suggests that the alveolar epithelium gets stimulated by the deposited particles only for a short period of time and then recovers to basal conditions. One reason explaining this dynamic inflammatory kinetic could be related to the rapid phagocytic particle clearance by resident AM, as no increase in the percentage of particle laden cells is observed 12 h after IT. In fact our data suggests that the pulmonary deposited CNPs can only develop their pro-inflammatory potency on the epithelium until engulfed by macrophages. From that point on the epithelial irritation can recover since CNP laden AMs do obviously not promote inflammation but rather remain inactive until eventually cleared by mucociliary removal. In vitro exposure of ATII cells to high doses carbon black nanoparticles has previously been shown to stimulate the release of macrophage chemoattractants [68], suggesting that structural cells might trigger the chemotaxis of AMs to the site of particle deposition. In this light the outdated term “dust cells” for alveolar macrophages highlights their specification in a way that for ‘low‐solubility low‐toxicity particles’ the essential function of AMs is limited to the removal of particles from the lining fluid to prevent epithelial irritation and inflammation, but not to trigger inflammation itself.

## Conclusions

We show that pulmonary carbon nanoparticle instillation triggers acute pulmonary inflammation, with no inflammatory stimulation of alveolar macrophages, but alveolar epithelial cells during the first 12 h after exposure,-the period of the maximal induction of pro-inflammatory gene expression. As alveolar deposited particles are rapidly removed by phagocytic clearance from the respiratory surface, the epithelial inflammatory response seems short-lived as long as particle laden macrophages remain unstimulated. Yet inhaled materials which are difficult to clear by phagocytosis or pose toxicity and persistent stimulation to alveolar macrophages, may cause persistent inflammation and should therefore be designated more hazardous than ‘low-toxicity low-solubility particles’ such as carbon nanoparticles.

## Methods

### Carbon nanoparticles

Carbon nanoparticles (CNP) were generated by spark discharge from graphite electrodes as previously described [47, 69]. The sterile and pyrogene-free produced particles consist of over 96 % pure carbon and primary particles are characterized by an isometric/spherical shape with a diameter of 7–12 nm and a specific surface area of 800 m^2^/g [12, 49]. CNPs were dispersed in pyrogene-free distilled water (Aqua ad iniectabilia, Braun, Melsungen, Germany) at a concentration of CNP 0.4 μg/μl and sonicated twice for 30 s with a probe sonicator (SonoPlus HD70, Bachofer, Berlin, Germany) at 30 % amplitude (70 W) on ice. CNP dispersions showed a mean agglomerate size of 0.19 μm, measured by dynamic light scattering (Malvern Zetasizer Nano ZS). The absence of endotoxins and pyrogens from the particle preparation was approved by LIMULUS assay and even more relevant by in vitro studies using different murine macrophage cell lines (data not shown).

### Animals

Female C57BL/6 J mice at the age of 8–10 weeks were received from Charles River Laboratories, Sulzfeld, Germany. Prior to the CNP exposure, the animals were kept for a minimum of two weeks in isolated ventilated cages (IVC-Racks; BioZone, Margate, UK) supplied with filtered air, in a 12-hr light/12-hr dark cycle at animal facility of Institute of Lung Biology and Diseases at the Helmholtz Zentrum München, for acclimatization. Specific pathogen-free hygienic status was approved by a health certificate according to Federation of Laboratory Animal Science Associations guidelines [70]. Food and water were available *ad libitum*. Animals were treated humanely and with regard for alleviation of suffering; experimental protocols were reviewed and approved by the Bavarian Animal Research Authority and by the Institutional Animal Care and Use Committee of the Helmholtz Center Munich.

### Nanoparticle exposure

Mice were exposed with either pyrogene-free distilled water (Aqua ad iniectabilia, Braun, Melsungen, Germany) for sham groups or 20 μg CNP in 50 μl water for experimental groups by intratracheal instillation as described previously [8–12]. Briefly, mice were anesthetized via intraperitoneal injection of a mixture of medetomidin (0.5 mg/kg bodyweight), midazolam (5 mg/kg bodyweight), and fentanyl (0.05 mg/kg bodyweight) and intubated by a nonsurgical technique, using a cannula inserted 10 mm into the trachea, for instillation. For the time course experiments of 3 h up to 7 days after exposure, each of the eight experimental groups consisted of six mice, and for the BAL cell isolation experiments, each of the four experimental groups consisted of five mice. For the lung cell isolation experiment, each of the four experimental groups consisted of three mice, and these experiments were performed independently for four times.

### BAL preparation and cell differentiation

3 h, 6 h, 12 h, 18 h, 1d, 3d and 7d, after CNP instillation, mice were anesthetized via intraperitoneal injection of a mixture of xylazine and ketamine and sacrificed by retrobulbar exsanguination. Immediately afterwards, BAL was performed by cannulating the trachea and infusing the lungs eight times with 1.0 ml PBS without calcium and magnesium, as described previously [12, 71]. BAL fluids of the first two lavages were pooled for protein and cytokine analysis after BAL cell separation by centrifugation (425 g, 20 min at room temperature). The cell pellets were suspended in 1 ml RPMI 1640 medium (BioChrome, Berlin, Germany) supplemented with 10 % fetal calf serum (Seromed, Berlin, Germany), and the number of living cells was counted by the trypan blue exclusion method. BAL cell differentials were performed on cytospin preparations (May-Grünwald- Giemsa staining; 2 × 200 cells counted) and the number of polymorphonuclear neutrophils (PMNs) was used as a marker of inflammation [12, 71].

### Enzyme-linked immunosorbent assay (ELISA)

BAL cytokine concentrations for TNFα, CXCL1, CXCL2 and CXCL5 were determined by commercial ELISAs (Mouse DuoSet ELISA, R&D Systems, Minneapolis, USA) according to the manufacturer’s instructions.

### Alveolar macrophages isolation

Alveolar macrophages were recovered from the lungs of mice by BAL with 8 washes of 1 ml PBS at room temperature. BAL cells were pelleted for 5 min at 425 g and washed twice in complete RPMI-1640 medium. 2 × 10^5^ cells were seeded in 12-well plates and were allowed to adhere for 2 h. Non-adherent cells were removed by washing two times with PBS.

### Alveolar epithelial and CD45+ cell isolation

Alveolar epithelial type II (ATII) cells and CD45+ lung leukocytes isolation has been described by Mutze and colleagues before [54, 72]. Briefly, animals where scarified 12 h after water or CNP instillation, and mouse lungs were lavaged twice with 500 μl of sterile PBS, then 0.9 % saline solution (B. Braun Melsungen AG, Melsungen, Germany) was flushed through the right heart to remove blood cells at best. Subsequently, lungs were inflated with 1.5 ml Dispase (BD Bioscience, San Jose, CA) and 300 μl of 1 % low melting point agarose (Sigma-Aldrich, Taufkirchen, Germany) and incubated for 45 min at room temperature. Lungs were minced and four lungs of each treatment condition were pooled to generate a single cell suspension by consecutively filtering the crude cell suspension through 100 μm, 20 μm, and 10 μm nylon meshes (Sefar, Heiden, Switzerland). Single cell suspension was centrifuged at 200 g for 10 min and the cell pellets were suspended in DMEM cell culture medium (Sigma Aldrich, Taufkirchen, Germany). Incubation of the single cell suspension on petri dishes coated with antibodies against CD45 and CD16/32 (both BD Bioscience, Heidelberg, Germany) for 30 min at 37 °C was performed to harvest macrophages and lymphocytes. Non-adherent cells were collected and negative selection for fibroblasts was performed by adherence for 25 min on cell culture dishes. Again, non-adherent cells were collected and cell viability was determined by trypan blue exclusion. Cell purity was assessed by immunofluorescence staining of cytospin preparations using antibodies for proSFTPC (Merck Millipore, Darmstadt, Germany), panCK (Dako, Hamburg, Germany), CD45 (BD Bioscience, Heidelberg, Germany), and αSMA (Sigma Aldrich, Taufkirchen, Germany). Finally, CD45+, alveolar epithelial and BAL cells were collected for RNA isolation.

### RNA isolation and quantitative real-time PCR (qRT-PCR) analysis

For gene expression profiling, lung tissues and BAL macrophages were prepared for total RNA isolation using the RNeasy Mini Kit (Qiagen, Hilden, Germany) according to the manufacturer’s instructions. Total RNA concentrations were analyzed by Nanodrop system (PEQLAB Biotechnologie GMBH, Erlangen, Germany), and for RNA quality confirmation the Agilent 2100 Bioanalyzer chip system (Agilent Technologies, Böblingen, Germany) with the Agilent RNA 6000 Kit were used. cDNA synthesis was performed using the SuperscriptII system (Invitrogen, Carlsbad, USA). Quantitative PCR was performed using SYBR green ROX mix (Thermo Fisher Scientific, Waltham, Massachusetts, USA), analyzed by the ABI Prism Sequence Determination System (Applied Biosystems, California, USA). Gene specific primers are listed in Table 1 and actin, beta (Actb) and hypoxanthine guanine phosphoribosyl transferase (Hprt) transcripts were used as the housekeeping genes for the standardization of the relative mRNA expressions. The detailed procedure has been described by Yin et al. [73].

**Table 1:** Primer sequences for qPCR analysis

Gene	MGI ID	5'-primer	3'-primer
Actb	MGI:87904	TCCATCATGAAGTGTGACGT	GAGCAATGATCTTGATCTTCAT
Hprt	MGI:96217	GTTGGATACAGGCCAGACTTTGT	CACAGGACTAGAACACCTGC
Cxcl1	MGI:108068	CCGAAGTCATAGCCACAC	GTGCCATCAGAGCAGTCT
Cxcl2	MGI:1340094	TCCAGAGCTTGAGTGTGACG	TCCAGGTCAGTTAGCCTTGC
Cxcl5	MGI:1096868	CCCTACGGTGGAAGTCAT	CTTCACTGGGGTCAGAGT
Nos2	MGI:97361	CCTGTGAGACCTTTGATG	CCTATATTGCTGTGGCTC
Tnf	MGI:104798	CACCACGCTCTTCTGTCTG	GCTACAGGCTTGTCACTC
Il1b	MGI:96543	CAACCAACAAGTGATATTCTCCATG	GATCCACACTCTCCAGCTGCA
Nfkbia	MGI:104741	TACTCCCCCTACCAGCTTAC	GACTCCGTGTCATAGCTCTC
Nfkbib	MGI:104752	CTGCATCTAGCAGCCATC	GGCTCTGAGTGAGGTAGGTA
Nfkbiz	MGI:1931595	GCCAGGTCCAGATTAGGT	CCTCACCTAGAGAGAGAGCA
Cd68	MGI:88342	CCTCCACCCTCGCCTAGTC	TTGGGTATAGGATTCGGATTTGA
Aqp1	MGI:103201	CATCACCTCCTCCCTAGTC	GCTGAGCCACCTAAGTCTC

### Immunofluorescence (IF) staining

For purity control of isolated pmATII cells, IF staining was performed on cells cultured overnight on chamber slides (BD Bioscience). Cells were subsequently fixed with acetone/methanol (1:1), and blocked with 3 % (w/vol) bovine serum albumin (Sigma Aldrich) for 30 min at room temperature (RT). Primary antibodies (proSFTPC (Merck Millipore, Darmstadt, Germany), panCK (Dako, Hamburg, Germany), T1α (Podoplanin; R&D Systems, Minneapolis, MN, US), CD45 (BD Bioscience), CD31 (Abcam, Cambridge, UK), αSMA (Sigma Aldrich)) were diluted in PBS containing 0.1 % (w/vol) BSA and incubated for 1 h at RT. Fluorescently labeled secondary antibodies (polyclonal goat anti-mouse FITC, Dako, Hamburg, Germany; goat anti-rabbit Alexa 555 or goat anti-rat Alexa 555, both Life Technologies) were diluted in PBS containing 0.1 % (w/vol) BSA and incubated for 1 h at RT. DAPI staining (Roche, Basel, Switzerland) was performed to visualize cell nuclei.

### Statistical analysis

All values are showed as the mean ± SEM of at least five animals or four independent samples for isolated cells. We used the analysis of variance (ANOVA), as calculated by GraphPad Prism 5, to determine the statistical significance of differences between the experimental groups. Individual inter-group comparisons were analysed using the two-tailed unpaired *t* test with Welch’s correction. Differences were considered significant at *, *p* < 0.05; **, *p* < 0.01 and ***, *p* < 0.001.

## Abbreviations

AM, alveolar macrophage; ATI, alveolar epithelial cell, type I; ATII, alveolar epithelial cell, type II; BAL, bronchoalveolar lavage; CNP, carbon nanoparticles; CXCL, chemokine (C-X-C motif) ligand; H&E, haematoxylin and eosin; IT, intratracheal instillation; PMN, polymorphonuclear neutrophils; RT-PCR, real-time polymerase chain reaction; SEM, standard error of the mean; TNFα, tumor necrosis factor alpha

## Additional file

Additional file 1: Additional Methods. **Figure S1.** Ultrastructure of CNP-aerosols (A) and aqueous dispersion (B). **Figure S2.** Enrichment of cell-specific gene expression in CD45- and CD45+ lung cells and total BAL cells. **Figure S3.** Purity of isolated primary murine ATII cells. **Figure S4.** Synopsis of gene expression results of all four cell isolation experiments. **Figure S5A.** Gene expression profile of by BAL macrophages 3 and 12 h after CNP and 12 h after LPS instillation. **Figure S5B.** BAL neutrophil and macrophage numbers 3 and 12 h after CNP and 12 h after LPS instillation. **Figure S6.** Detection of inflammatory cell activation by immunostaining, we used NFkB-GFP-reporter mice. Additional Reference. (PDF 541 kb)
