# Supplementary material for: No involvement of alveolar macrophages in the initiation of carbon nanoparticle induced acute lung inflammation in mice
Source: Part Fibre Toxicol. 2016 Jun 21;13:33. doi: 10.1186/s12989-016-0144-6 (PMC4915176; doi:10.1186/s12989-016-0144-6)
Supplement: Additional file 1: — Additional Methods. Figure S1. Ultrastructure of CNP-aerosols (A) and aqueous dispersion (B). Figure S2. Enrichment of cell-specific gene expression in CD45- and CD45+ lung cells and total BAL cells. Figure S3. Purity of isolated primary murine ATII cells. Figure S4. Synopsis of gene expression results of all four cell isolation experiments. Figure S5A. Gene expression profile of by BAL macrophages 3 and 12 h after CNP and 12 h after LPS instillation. Figure S5B. BAL neutrophil and macrophage numbers 3 and 12 h after CNP and 12 h after LPS instillation. Figure S6. Detection of inflammatory cell activation by immunostaining, we used NFkB-GFP-reporter mice. Additional Reference. (PDF 541 kb) [file 12989_2016_144_MOESM1_ESM.pdf]

Supplement:

No involvement of alveolar macrophages in the initiation of carbon nanoparticle induced acute lung inflammation in mice

Shanze Chen[1, 2*], Renfu Yin[1, 3*], Kathrin Mutze[1], Youjia Yu[1], Shinji Takenaka[1], Melanie Königshoff[1], Tobias Stoeger[1#]

List of Additional Files:

Additional Methods:

Figure S1: Ultrastructure of CNP-aerosols (A) and aqueous dispersion (B).

Figure S2: Enrichment of cell-specific gene expression in CD45- and CD45+ lung cells and total BAL cells

Figure S3: Purity of isolated primary murine ATII cells.

Figure S4: Synopsis of gene expression results of all four cell isolation experiments

Figure S5A: Gene expression profile of by BAL macrophages 3 and 12h after CNP and 12h after LPS instillation

Figure S5 B: BAL neutrophil and macrophage numbers 3 and 12h after CNP and 12h after LPS instillation

Figure S6: Detection of inflammatory cell activation by immunostaining, we used NFkB-GFP-reporter mice

Additional Reference

Additional Methods:

CNP generation

Particles were generated by the method described by Roth et al. 2004. The aerosol consists of primary particles of 7-12nm diameter, forming agglomerates with an average number size distribution of 48.9 ±1.8 nm (equivalent mobility diameter) and a mean number concentration of 7.7 ±0.86 10^{6} cm^{-3} (Andre et al. 2006). According to the particle spectra, 92% of the generated particle agglomerates where nanoparticles (<0.1 µm). Airborne particles were collected on 0.2 µm pore size polytetrafluorethylen (PTFE) filters (no. 11807–50-N, Sartorius, Germany) using a vacuum pump, and removed from the filters using a stainless steel spatula in a clean bench (Figure S1A). Particles were resupended in water in the same way as for the instillation procedure, mounted on the TEM-grids and analyzed by transmission electron microscopy (TEM) as described previously (Stoeger at al. 2006). CNP dispersions showed a mean agglomerate size of 0.19 µm, measured by dynamic light scattering (Malvern Zetasizer Nano ZS).

Immunostaining

Briefly, lungs were embedded in paraffin and sectioned (5 µm). After deparaffinization (xylene and gradual ethanol), slides were incubated in H_2O_2 buffer (40ml Methanol, 3ml 30% H_2O_2, 7ml ddH_2O) for 20 min at room temperature followed by antigen retrieval at 125°C for 15min. After blocking sections were incubated with rabbit polyclonal anti GFP antibody (1:2000, ab290, Abcam, USA) at 4°C overnight and alkaline phosphatase conjugated goat anti-rabbit IgG as secondary antibody at room temperature for 30min. Detection was done using the Vulcan Fast Red Chromogen Kit 2 (901-FR805-080515, Biocare Medical, USA) according to the manufacturer's instructions.

Figure S1:

A **B**

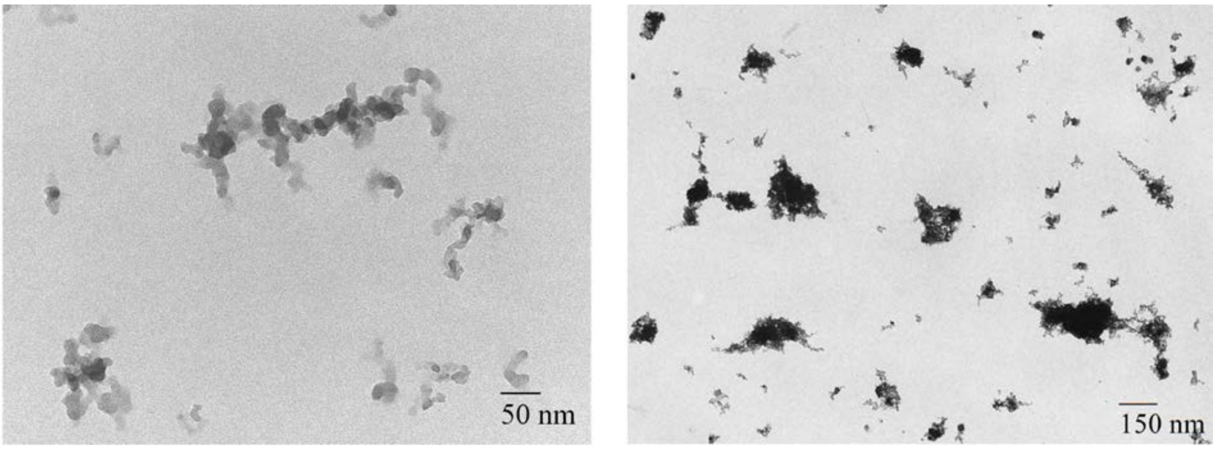

Figure S1: Ultrastructure of CNP-aerosols (A) and aqueous dispersion (B).

Figure S2:

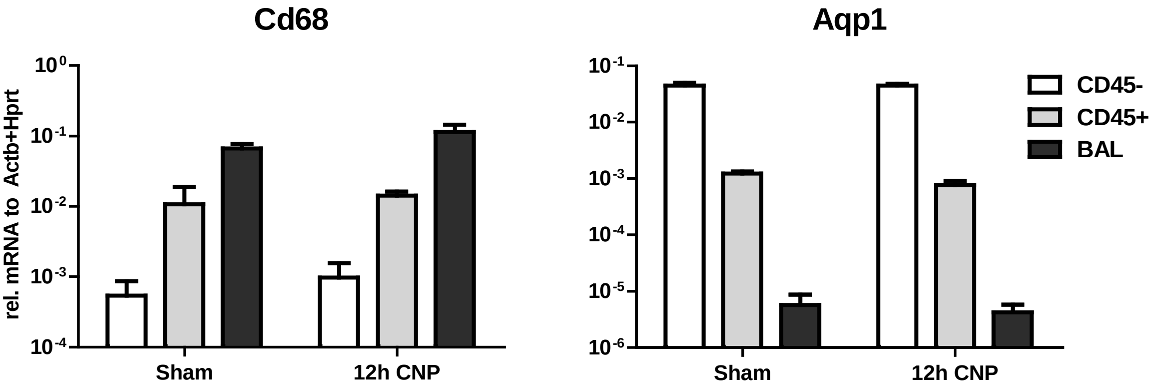

Figure 2S shows the enrichment of macrophage-specific Cd68 and alveolar epithelial cell-specific Aqp1 gene expression in CD45- and CD45+ lung cells and total BAL cells. Expression levels are given at the log-10 scale, relative to Actb + Hprt. Results show means and SEM out of 3 replicas and the expression pattern are representative for three independent experiments.

Figure S3

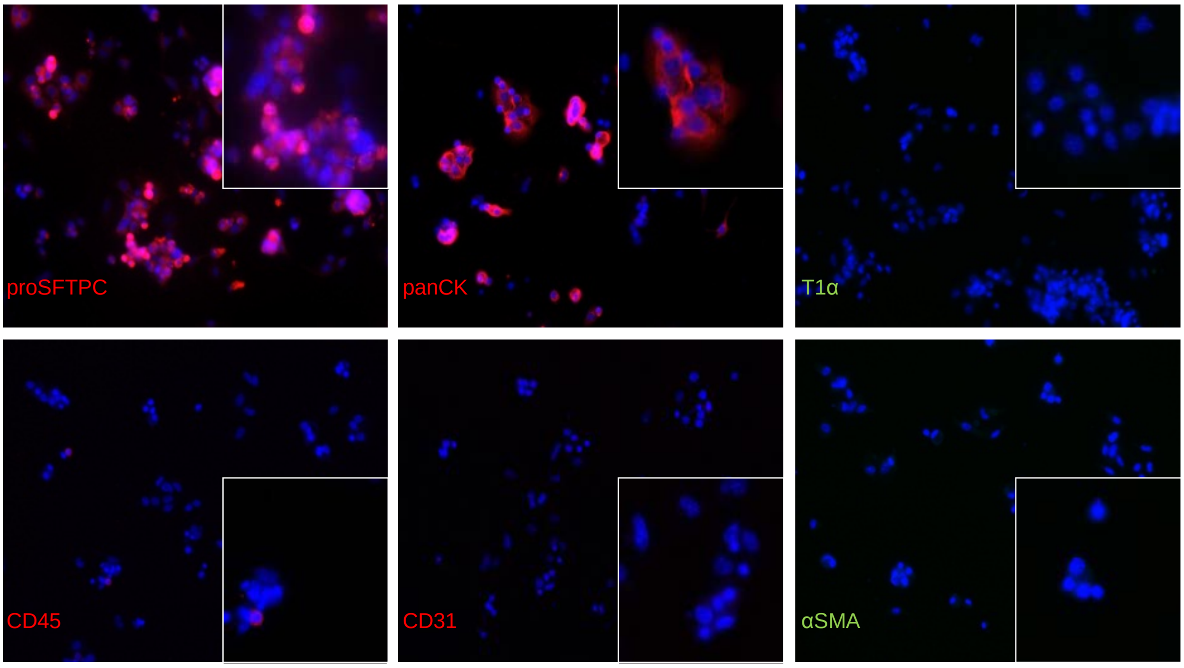

Figure S3: Purity of isolated primary murine ATII cells. Immunofluorescence staining was performed on primary murine ATII cells cultured on chamber slides over night for assessment of purity. Fluorescent images represent a 200 x magnification, whereas image inserts represent a 400 x magnification. Stainings are shown in red for the ATII cell marker proSFTPC (pro surfactant protein C) and panCK (Pan Cytokeratin), the endothelial marker CD31, and the leucocyte marker CD45, as well as in green for the ATI cell marker T1α (podoplanin) and the fibroblast marker α-Sma (α-smooth muscle actin).

Figure S4:

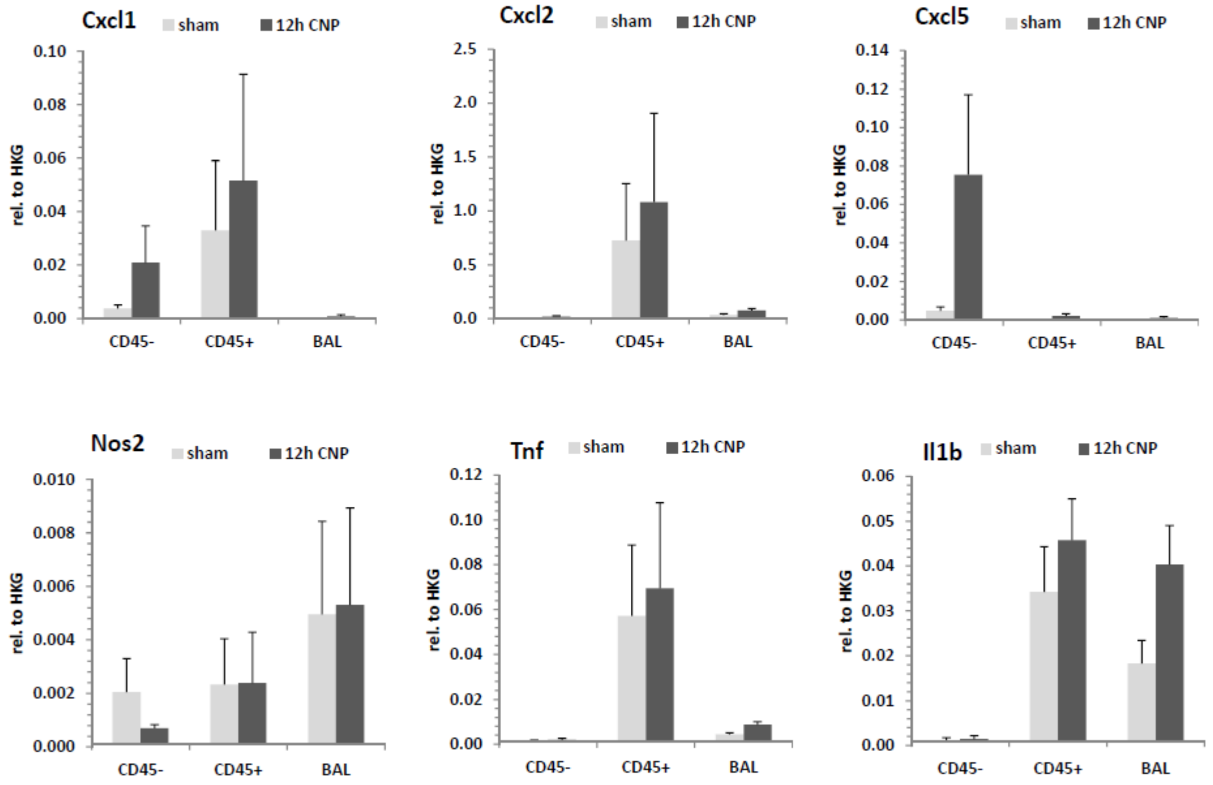

Figure S4 shows a synopsis of the gene expression results from the four individual cell isolation experiments, of which one representative profile is given in figure 6. CD45- (alveolar epithelial cells), CD45+ leukocytes and total BAL cells were isolated from mice 12 h after the treatment with water (sham) or CNP. Expression levels are given relative to Actb + Hprt. Results show means and SEM out of four independent experiments.

Even if the inter-experimental variation of expression signals caused considerable standard errors which mask statistical significance, CNP triggered inductions still get evident for the genes Cxcl1 and -5. Note that the CNP exposure related changes for Tnf and Il1b expression in BAL cell isolates shown here, are absent in figure 5 where macrophages purified from BAL cells have been used (see METHODS: “Alveolar macrophages isolation”). Since limitations in BAL cell numbers did not allow for AM purification, the BAL cell expressions shown in Figure

S4 are besides alveolar macrophages also derived from neutrophil granulocytes, thereby distorting the macrophage related expression pattern shown in figure 5.

Figure S5A

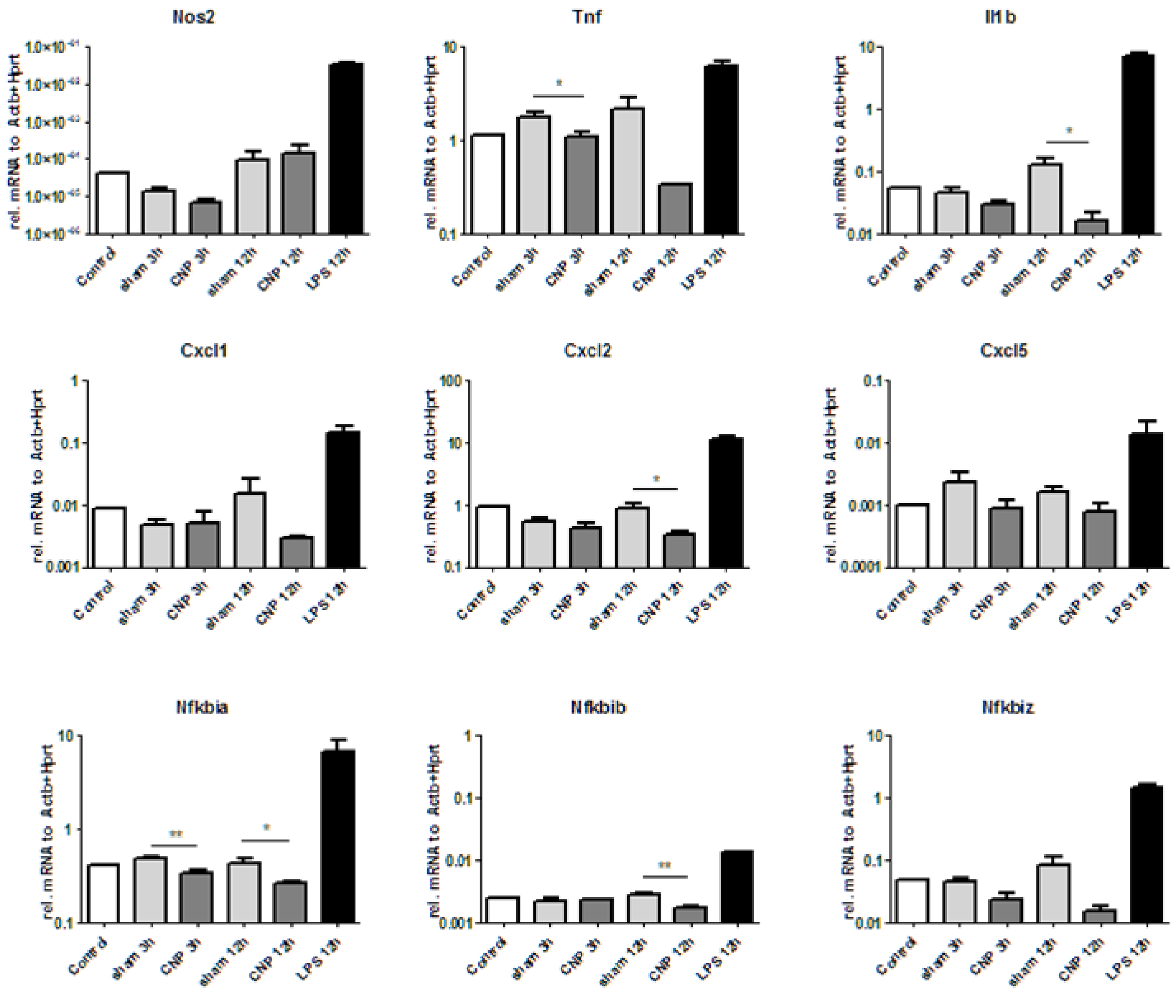

Figure S5A shows the gene expression profile of by BAL isolated and attachment purified macrophages, similar as investigated in Figure 5. In this independent round of experiment, mice were left untreated (control, n = 2), instilled with water (sham; n = 6), or again 20ug CNP (n = 6) or 0.1ug LPS (E. coli O55:B5, strain CDC 1644–70; n = 5) as positive control for macrophage activation. BAL was performed after 3 and 12h for the sham and CNP groups

and 12h for the LPS positive control. Gene expression is shown for of the macrophage activation markers Nos2, Tnf and Il1b (upmost row), the chemokines Cxcl1, Cxcl2 and Cxcl5 (middle row) and the NF- κB signaling pathway genes Nfkbia, Nfkbib and Nfkbiz (lowest row) and presented relative to Actb & Hprt. Values are given as mean ± SEM, asterisks represent significance as compared to respective sham group with * p<0.05, and **p<0.01.

Figure S5B

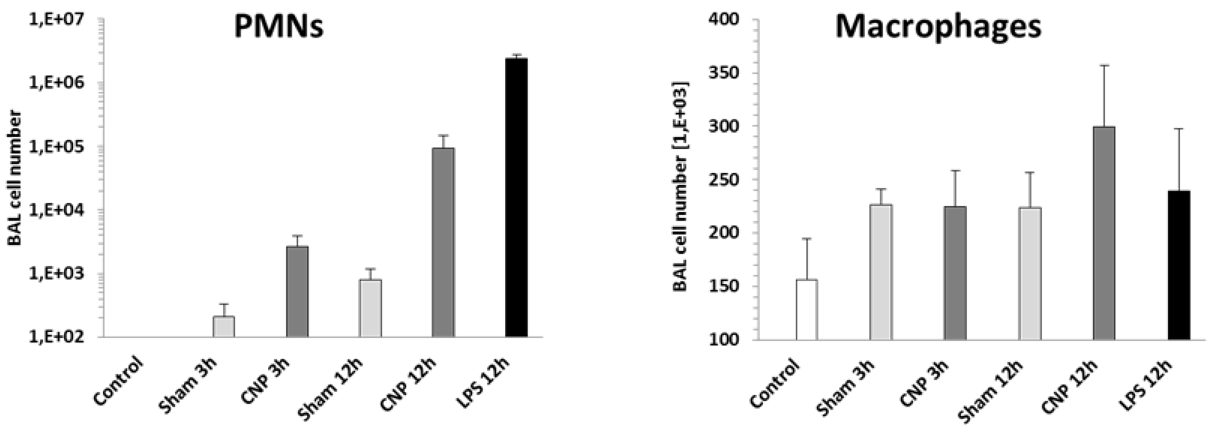

Figure S5B illustrates the inflammatory response, shown by the BAL neutrophil (PMN) and macrophage numbers. Because of the wide spectrum of the inflammatory response, PMN numbers are blotted at the logarithmic scale.

Figure S6

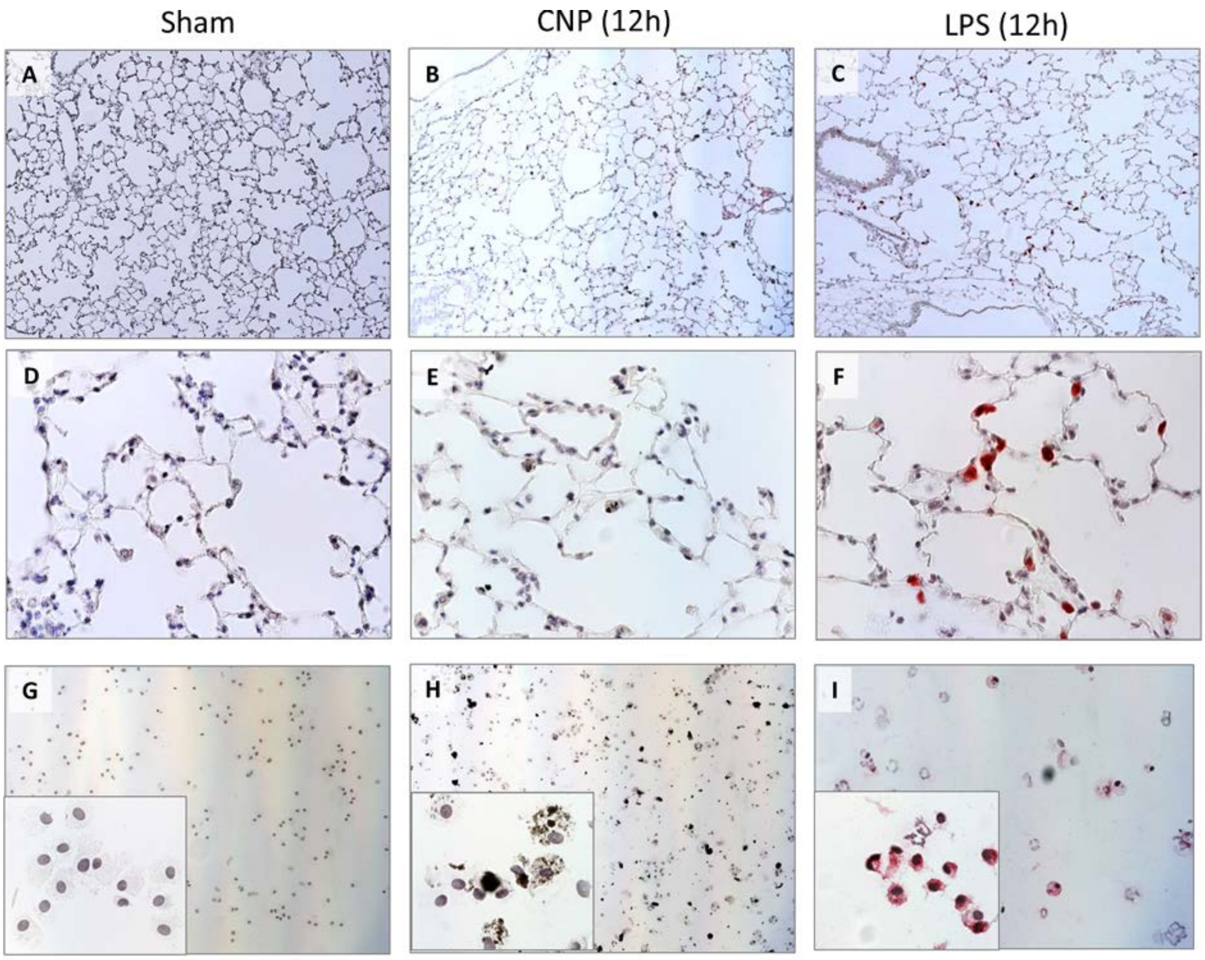

Figure S6: To detect inflammatory cell activation at the histological level by immunostaining, we used NFkB-GFP-reporter mice (cis-NF-kBEGFP, C57BL/6 strain, Magness et al. *J Immunol.* 2004; three NF-kB cis elements drive enhanced GFP). The NF-kappa-B1 driven GFP-reporter protein was detected on PFA (4%) fixed lungs (A - F) or cytospins (G – I) with the rabbit polyclonal anti-GFP antibody. Mice were intratracheally exposed to 50 ul water (sham: A, D, G), 20ug CNP (B, E, H) or 0.1ug LPS (C, F, I) for 12h. Figure S61 D, E and F show a 4 fold magnification of A, B and C. Immunostaining for the NFkB driven GFP-reporter protein shows a clear inflammatory activation of alveolar epithelial cells and macrophages (red staining) for the LPS exposed lungs and BAL macrophages, not seen in sham and CNP exposed animals. Images shown are representative for micrographs obtained from two different mice.

Additional Reference:

Magness ST, Jijon H, Van Houten Fisher N, Sharpless NE, Brenner DA, Jobin C. In vivo pattern of lipopolysaccharide and anti-CD3-induced NF-kappa B activation using a novel gene-targeted enhanced GFP reporter gene mouse. *J Immunol.* 2004 Aug 1;173(3):1561-70
